# Response to Edaphoclimatic Conditions and Crop Management of the Bacterial Microbiome of Musa acuminata Rhizosphere Profiled by 16S rRNA Gene Amplicon Sequencing

**DOI:** 10.1128/MRA.01437-20

**Published:** 2021-03-11

**Authors:** Francisco J. De la Torre-González, Elisa Fernández-Castillo, Dailen Azaharez-Llorente, Jorge Lara, Enrique Avendaño, Alexis Castañeda, Sergio Gómez, José Gaxiola, Ali Asaff-Torres, Jorge Verdín

**Affiliations:** a Innovak Global, Chihuahua, Mexico; b CIAD-Centro de Investigación en Alimentación y Desarrollo, A.C., Hermosillo, Sonora, Mexico; c Grupo Bananero Mi estrella “E”, Plantación Sonora, Teapa, Tabasco, Mexico; d CIATEJ-Centro de Investigación y Asistencia en Tecnología y Diseño del Estado de Jalisco, A.C., Zapopan, Jalisco, Mexico; Indiana University, Bloomington

## Abstract

Bacterial rhizospheric microbiomes of Musa acuminata cultivated in farms close to the west and east Mexican coasts and with different climate, soils, and crop management practices were characterized by 16S rRNA gene amplicon sequencing. Results showed that rhizospheric microbiome composition changed along with seasonal weather but were mostly indifferent to soil type.

## Abstract (precis)

Bacterial rhizospheric microbiomes of Musa acuminata cultivated in farms close to the west and east Mexican coasts and with different climate, soils, and crop management practices, were characterized by 16S rRNA gene amplicon sequencing. Results showed that rhizospheric microbiome composition changed along with seasonal weather but were mostly indifferent to soil type.

## ANNOUNCEMENT

Banana (*Musa* spp.) is one of the most produced crops in the world (1). The rhizosphere and endosphere microbiomes of *Musa* spp. have been intensely investigated to address the major threat to banana production, *Fusarium* wilt (2–7). Nevertheless, the dynamic behavior of such microbiomes throughout seasons in different climates, soil types, and crop management practices have not been analyzed, despite the fact that they may provide valuable information to prevent diseases or increase productivity. Here, we report the 16S rRNA gene profiling of the Musa acuminata rhizosphere cultivated in two climate regimes, different soil types, and different types of crop management, which include the addition of biostimulants.

Bulk soil and roots of banana plants conventionally cultivated with or without microbial biostimulants (BioFit RTU and Mycoroot, Innovak, Mexico; 1 kg BioFit RTU + 1 kg Mycoroot/200 liters water/ha, three 4-month-spaced applications during the cropping year; 2 kg Mycoroot/200 liters water/ha, 2 weeks after each dual application) were sampled (random blocks) in three farms from southern Mexico close to the east and west coasts—plantation SB, Chiapas (14°54′25″N, 92°26′26″W; average temperature, 26.3°C; average annual rainfall, 2,158 mm), and plantations SO and RE, Tabasco (17°37′31″N, 92°57′05″W; average temperature, 26.9°C; average annual rainfall, 3,862 mm) (8, 9). The Chiapas samples were collected only during December 2017, while samples from Tabasco were collected during February, June, and December 2018. In addition, the Tabasco samples were collected from banana cultivated in three soil types, sandy loam, clay loam, and silty loam. All samples were triplicated, each one composed of seven plant roots (collected during the inflorescence emission of the mother plant) and seven 20-cm-deep soil columns for rhizosphere and bulk soil samples.

For rhizospheric DNA extraction, excess soil on the roots was mechanically removed. Then, the roots were washed in 200 ml sterile phosphate-buffered saline (PBS)-Silwet Maxx (Arysta LifeScience, Mexico) (0.02% [vol/vol]) on sterile bottles and shaken at 250 rpm, 4°C for 20 min. Afterward, washed roots were taken off and submerged again in PBS-Silwet Maxx under sterile conditions, shaken at 250 rpm at 4°C for 20 min, and sonicated (VCX-130PB Ultrasonic Processor; Fisher Scientific) at 70% frequency for 5 min. The rhizosphere fraction was recovered by centrifugation (3,857 × *g*, 4°C, 20 min) and kept at −80°C until metagenomic DNA extraction. Bulk soil and rhizosphere (250 mg) metagenomic DNA were extracted with a DNeasy PowerSoil kit (Qiagen, Germany) following the manufacturer’s instructions, and after extraction, its integrity and concentration were assessed by agarose gel electrophoresis and UV spectroscopy.

16S V3-V4 rRNA was amplified with 337F/805R primers (25 PCR cycles) and indexed with a Nextera XT index kit v2 (Illumina) (8 PCR cycles) using Phusion (Thermo Fisher) DNA polymerase (10). 16S rRNA amplicon libraries were paired-end sequenced on an Illumina MiSeq platform. A total of 25,675,772 raw reads were obtained for 16S libraries (Table 1). Sequencing reads were analyzed with CLC Genomics Workbench 9.0 and CLC Microbial Genomics module 1.3 (Qiagen, Denmark). Raw reads were overlapped into single longer reads and fixed-length trimmed; chimeras and reads showing <100 abundance were removed. To identify operational taxonomic units (OTUs), filtered reads were clustered against the SILVA 16S database 138.1 (11) using 97% identity as clustering criteria. A total of 79,182 OTUs were predicted for 16S libraries.

**TABLE 1:** Characteristics and SRA accession numbers of 16S rRNA sequences obtained in this study

Location	Soil type	Sample type	Sampling date (mo/yr)	Sample name	SRA accession no. for^*a*^:		
					Rep 1	Rep 2	Rep 3
Chiapas SB	Loamy	Bulk soil	12/2017	16S bulk soil Chiapas Rep 1, 2, 3	SRR12963519	SRR12963518	SRR12963562
		Rhizosphere-control	12/2017	16S rhizosphere Chiapas Rep 2, 3		SRR12963551	SRR12963540
		Rhizosphere-biostimulant	12/2017	16S rhizosphere Chiapas Biostimulant, Rep 1, 2, 3	SRR13237956	SRR13237955	SRR13237944
Tabasco SO	Sandy loam	Bulk soil	2/2018	16S bulk soil tab sandy T1 Rep 1, 2, 3	SRR12963529	SRR12963523	SRR12963522
		Rhizosphere-control	2/2018	16S rhizosphere tab sandy T1 Rep 2, 3		SRR12963521	SRR12963520
		Rhizosphere-biostimulant	2/2018	16S rhizosphere tab sandy T1 Biostimulant, rep 1, 2, 3	SRR13237935	SRR13237934	SRR13237933
		Bulk soil	6/2018	16S bulk soil tab sandy T2 Rep 1, 2, 3	SRR12963559	SRR12963558	SRR12963557
		Rhizosphere-control	6/2018	16S rhizosphere tab sandy T2 Rep 1, 2, 3	SRR12963556	SRR12963555	SRR12963554
		Rhizosphere-biostimulant	6/2018	16S rhizosphere tab sandy T2 Biostimulant, Rep 1, 2, 3	SRR13237954	SRR13237953	SRR13237952
		Bulk soil	12/2018	16S bulk soil tab sandy T3 Rep 1, 2, 3	SRR12963543	SRR12963542	SRR12963541
		Rhizosphere-control	12/2018	16S rhizosphere tab sandy T3 Rep 1, 2, 3	SRR12963539	SRR12963538	SRR12963537
		Rhizosphere-biostimulant	12/2018	16S rhizosphere tab sandy T3 Biostimulant, Rep 1, 2, 3	SRR13237945	SRR13237943	SRR13237942
Tabasco SO	Clay loam	Bulk soil	2/2018	16S bulk soil tab clay T1 Rep 1, 2, 3	SRR12963517	SRR12963516	SRR12963570
		Rhizosphere-control	2/2018	16S rhizosphere tab clay T1 Rep 1, 2, 3	SRR12963569	SRR12963568	SRR12963567
		Rhizosphere-biostimulant	2/2018	16S rhizosphere tab clay T1-biostimulant, rep 1, 2	SRR13237932	SRR13237931	
		Bulk soil	6/2018	16S bulk soil tab clay T2 Rep 1, 2, 3	SRR12963553	SRR12963552	SRR12963550
		Rhizosphere-control	6/2018	16S rhizosphere tab clay T2 Rep 1, 2, 3	SRR12963549	SRR12963548	SRR12963547
		Rhizosphere-biostimulant	6/2018	16S rhizosphere tab clay T2 Biostimulant, rep 1, 2, 3	SRR13237951	SRR13237950	SRR13237949
		Bulk soil	12/2018	16S bulk soil tab clay T3 Rep 1, 2, 3	SRR12963536	SRR12963535	SRR12963534
		Rhizosphere-control	12/2018	16S rhizosphere tab clay T3 Rep 1, 2, 3	SRR12963533	SRR12963532	SRR12963531
		Rhizosphere-biostimulant	12/2018	16S rhizosphere tab clay T3 Biostimulant, rep 1, 2, 3	SRR13237941	SRR13237940	SRsR13237939
Tabasco SO	Silty loam	Bulk soil	2/2018	16S bulk soil tab silty T1 Rep 1, 2, 3	SRR12963566	SRR12963565	SRR12963564
		Rhizosphere-control	2/2018	16S rhizosphere tab silty T1 Rep 1, 2, 3	SRR12963563	SRR12963561	SRR12963560
		Rhizosphere-biostimulant	2/2018	16S rhizosphere tab silty T1 Biostimulant, rep 1, 2	SRR13237930	SRR13237929	
		Bulk soil	6/2018	16S bulk soil tab silty T2 Rep 1	SRR12963546		
		Rhizosphere-control	6/2018	16S rhizosphere tab silty T2 Rep 1, 2	SRR12963545	SRR12963544	
		Rhizosphere-biostimulant	6/2018	16S rhizosphere tab silty T2 Biostimulant, rep 1, 2, 3	SRR13237948	SRR13237947	SRR13237946
		Bulk soil	12/2018	16S bulk soil tab silty T3 Rep 1, 2, 3	SRR12963530	SRR12963528	SRR12963527
		Rhizosphere-control	12/2018	16S rhizosphere tab silty T3 Rep 1, 2, 3	SRR12963526	SRR12963525	SRR12963524
		Rhizosphere-biostimulant	12/2018	16S rhizosphere tab silty T3 Biostimulant, rep 1, 2, 3	SRR13237938	SRR13237937	SRR13237936
Tabasco RE	Silty loam	Bulk soil	2/2018	16S bulk soil tab RE T1 Rep 1, 2, 3	SRR13234470	SRR13234469	SRR13234458
		Rhizosphere-control	2/2018	16S rhizosphere tab RE T1 Rep 1, 2, 3	SRR13234450	SRR13234449	SRR13234448
		Rhizosphere-biostimulant	2/2018	16S rhizosphere tab RE T1 Biostimulant, rep 1, 2, 3	SRR13234447	SRR13234446	SRR13234445
		Bulk soil	6/2018	16S bulk soil tab RE T2 Rep 1, 2, 3	SRR13234444	SRR13234468	SRR13234467
		Rhizosphere-control	6/2018	16S rhizosphere tab RE T2 Rep 1, 2, 3	SRR13234466	SRR13234465	SRR13234464
		Rhizosphere-biostimulant	6/2018	16S rhizosphere tab RE T2 Biostimulant, rep 1, 2, 3	SRR13234463	SRR13234462	SRR13234461
		Bulk soil	12/2018	16S bulk soil tab RE T3 Rep 1, 2, 3	SRR13234460	SRR13234459	SRR13234457
		Rhizosphere-control	12/2018	16S rhizosphere tab RE T3 Rep 1, 2, 3	SRR13234456	SRR13234455	SRR13234454
		Rhizosphere-biostimulant	12/2018	16S rhizosphere tab RE T3 Biostimulant, rep 1, 2, 3	SRR13234453	SRR13234452	SRR13234451

a Rep, replicate.

Rhizosphere microbiomes were different from those of their surrounding bulk soil but derived from them. Bulk soil and rhizosphere microbiome composition changed along with seasonal weather and, in some cases, also after biostimulant application; however, soil type did not show any influence.

### Data availability.

The sequences obtained in this study were made public in the Sequence Read Archive (SRA) (accession numbers are listed in Table 1) via the National Center for Biotechnology Information (NCBI) under the accession number PRJNA673638.
